# SeRUN^®^ study: Development of running profiles using a mixed methods analysis

**DOI:** 10.1371/journal.pone.0200389

**Published:** 2018-07-10

**Authors:** Manuela Besomi, Jaime Leppe, Maria Cristina Di Silvestre, Jenny Setchell

**Affiliations:** 1 School of Physical Therapy, Universidad del Desarrollo, Santiago, Chile; 2 School of Health and Rehabilitation Sciences, University of Queensland, Brisbane, Queensland, Australia; University of Illinois at Urbana-Champaign, UNITED STATES

## Abstract

**Objective:**

To determine profiles of urban runners based on socio-demographic, health, motivational, training characteristics and running-related beliefs and behaviours.

**Methods:**

Mixed, exploratory, sequential study with two stages: 1) quantitative, using an online survey; and 2) qualitative, using semi-structured interviews with runners from the previous stage. Participants were recruited via: running routes commonly attended by runners, eight races, previous databases and social media networks. The survey collected information on six dimensions: (1) socio-demographic; (2) health; (3) motivations; (4) training characteristics; (5) running-related behaviour; and (6) beliefs and perceptions about health. Profiles were identified using a two-step hierarchical clustering analysis. Subsequently, 15 interviews were conducted with participating runners across each of the identified profiles. Qualitative analysis complemented the profiles characterization, explaining motivations to start and continue running, beliefs about risk factors and injury prevention, and the physical therapist’s role in rehabilitation. Statistical analysis from stage one was conducted using SPSS 22 with a confidence level of 5%. Qualitative data were analysed using thematic and content analyses.

**Results:**

A total of 821 surveys were analysed (46% female), mean aged 36.6±10.0 years. Cluster analysis delineated four profiles (n = 752) according to years of running experience, weekly running volume and hours of weekly training. Profiles were named “Beginner” (n = 163); “Basic” (n = 164); “Middle” (n = 160) and “Advanced” (n = 265). Profiles were statistically different according to sex, age, years of running experience, training characteristics, previous injuries and use of technological devices (p<0.05). There were identified motivations to start and continue running. Beliefs about risk factors vary among stretching, footwear, training surface and overload. Runners identified the physical therapist as a specialist, involved in the rehabilitation process and showing empathy towards the patient. The identification of these profiles allows the generation of future prospective studies and clinical trials to evaluate risk and prognostic factors targeting specific populations of runners, with the ultimate aim of reducing running-related injury.

## Introduction

Running is the third most common physical activity (11.9%), after soccer (26.1%) and fitness (13.5%), among Chilean population [1]. The overall Running-Related Injuries (RRIs) incidence varies from 19.4% to 79.3%. These injuries are often related to musculoskeletal structural overload [2]. Although many injury prevention programs have been developed, particularly for novice and recreational runners, they have been largely ineffective in decreasing the incidence of RRIs [3–6]. The lack of consideration of intrinsic and extrinsic factors that define the behaviours of groups of runners could be an explanation of its inefficacy.

Kluitenberg et al. [7] described the prevalence of RRIs among different population of runners, classifying them according to their training or competition performance into: track (short, medium and long distance), novice, recreational, road long-distance, marathon, ultra-marathon, and cross-country runners. These criteria form groups that are not mutually exclusive, and subjects can be classified in more than one category (i.e. novice and road long distance runner), overlapping groups and potentially confusing the prevalence of RRIs. To the authors’ knowledge, no grouping classification of runners exists. Such grouping might help in future injury management strategies and research, as it has proved to be effective in other musculoskeletal conditions (low back pain) [8].

Different dimensions have been explored in association with RRIs, such as training factors [9–12], previous experience [13], motivations and health-related factors [14, 15]. These characteristics could define and explain certain risky behaviours [16], enabling the development of groups and profiles of runners for subsequent study on injury risk prevention. The inclusion of runners based only on their competitive status into ‘recreational’ or ‘competitive’ runners, might not allow the discrimination of suitable groups [13].

A model focused on the user's needs might enhance the management of RRIs [17, 18]. Mixed methods studies, which combine quantitative and qualitative data, offer a more global and integrated perspective of runners’ experiences of their musculoskeletal health [18]. The current study aims to determine profiles of urban runners, based on socio-demographics, health, motivational, training characteristics, and running-related beliefs and behaviours, through a mixed method design.

## Material and methods

### Study design and setting

This study used a sequential explanatory mixed methods approach, with a first quantitative and a second qualitative stage [19] Recruitment was carried out within the Metropolitan Region, Chile, in four main sites: thirty running routes, eight races, previous databases and social media networks. Runners were recruited by collecting their emails or through the official website (serun.udd.cl), during December 2015 and January 2016. A self-reported web-based cross-sectional survey was delivered during the quantitative first stage in order to develop running profiles from the quantitative data. The survey was made available during a 2-month period and collected information on six dimensions: 1) socio-demographic; 2) health; 3) training; 4) motivations; 5) behaviours associated to the practice of running; and 6) beliefs and perceptions about health. The subsequent qualitative stage consisted of semi-structured interviews with runners who participated in the first stage to expand understandings of the beliefs and behaviours particular to each profile.

### Participants

People who self-identified as being runners (with or without competitive goals) and who lived in Santiago—Chile were included, and those below 18 years old were excluded. Informed consent for stage one was implied by accepting the invitation to participate in the survey, which was included as an annex on the official website of the project (serun.udd.cl). In the qualitative stage, 15 subjects were recruited and voluntarily decided to continue to stage two between April and May 2016. All these participants read and signed an informed consent form in order to participate. This study was approved by the Scientific Ethics Committee at the Universidad de Desarrollo (Act of Approval 2016–76).

### Study size

In order to identify at least a 50% of novice or recreational runners [7], considering a confidence level of 95%, precision of 5% and an unknown population, 384 runners were needed for the quantitative stage. Considering differences by gender, at least 758 subjects were required. Sampling technique was selected based on convenience.

After identifying the running profile, the qualitative stage consisted in 15 semi-structured interviews with runners who had participated in the previous stage, keeping a proportional representation of gender and age between profiles. Of the participants who agreed to continue to this second stage of the study provided their email addresses for further contact. Runners were randomly selected from the previous stage sample, and relative saturation was required to reach sufficient findings to explain the complexity of the phenomena. Although the sampling technique was a convenience sample for both stages, the recruitment was intentionally performed in different environments with potential participants who varied in socioeconomic status, gender and age, as well as the type of running circuit (e.g. route, public park and private place).

### Data sources/Measurement

During the quantitative stage, the online survey was used to collect the information across six dimensions, with an average completion time of ten minutes. Socio-demographic dimensions included: education level, sex, age and marital status. Information about health included: body mass index (BMI), previous running-related injuries in the last 12 months [20], topography of previous injury, injury management, number of physical therapy sessions, hours of sleeping, hydration and eating habits. Motivational characteristics were: motivation for running and barriers / facilitators to run. Behaviours associated with running practice included: years of running experience, participation in competition, training surface, participation in other sports, technological devices, and associated costs to running (in Chilean Pesos). Training dimensions comprised of training program (self-administered or under professional supervision), frequency (days per week), weekly running volume (km/week), time dedicated per session (hours per session), physical preparation, and stretching. Beliefs and perceptions of runners included: quality of physical therapy treatment, self-classification as runner according to Kluitenberg et al. (i.e. novice, recreational, track, road long distance, marathon, ultra-marathon or cross-country runner) [7], and beliefs about the main reasons for injury and how to prevent it.

During the qualitative stage, a semi-structured interview guide was created (pilot tested with three runners) incorporating 14 questions grouped into four sections: 1) introduction: 2) running practice; 3) motivations for running; and 4) health/lifestyle and physiotherapist role. The detailed outline form is provided as a supplementary material (S1 File). All interviews were conducted face-to-face by three professionals trained in qualitative research (sociologists; 2 females and 1 male) who were independent of the primary research team (MB and JL). Interviewers were aware of the research aims but did not participate in further analyses. No previous relationship or interaction was established between the participant and the interviewer, and data was collected in a convenient place for the participant (e.g. workplace, cafe, training place). The duration of semi-structured interviews ranged between 25 and 35 minutes. A *posteriori* quality assessment tool for reporting qualitative research (COREQ-32) was used (S2 File) [21].

### Statistical methods

#### Quantitative analysis

A Shapiro-Wilk test was used to evaluate if all data for all quantitative variables were normally distributed. Descriptive analysis was presented with mean and standard deviation for quantitative variables. Nominal and ordinal qualitative variables were presented with absolute frequency and percentage. Sex differences and association with variables of interest for each dimension were analysed using separate two-tailed *t*-test and chi-square test, respectively.

The running profiles (groups) were developed through the following stages: 1) selection of variables according to expert criteria and evidence-based; 2) collinearity analysis using a Variance Inflation Factor (VIF) to assess multicollinearity among the selected variables in the previous stage; and 3) two-step hierarchical clustering analysis using Bayesian Information Criteria for grouping and discriminant analysis.

Variables in the model with a VIF higher than 2 points show a multicollinearity problem [22, 23]. This analysis was made to ensure that variables were not correlated (independent), so they do not affect the final clustering analysis. The primary purpose of cluster analysis is to add individuals based on their characteristics, forming groups with greatest possible internal homogeneity (within groups) and greatest possible external heterogeneity (between groups) [24]. The algorithm employed in the two-step cluster analysis can handle both categorical and continuous variables in the same model, and it automatically selects the best number of clusters according to the grouping criterion specified. This criterion of grouping was made according to the Bayesian Information Criterion (BIC) or Schwarz criterion, using a spontaneous classification for deciding the number of groups, and the model with the lowest BIC was preferred [25]. The discriminant analysis can be used in conjunction with the cluster analysis to confirm the results obtained in the cluster analysis, validating the employed grouping methodology [26].

In order to compare age, BMI, motivations, years of experience, training plan, associated costs, frequency, volume and hours of running training between profiles, ANOVA and chi-square test were used for quantitative and qualitative variables, respectively, and a Bonferroni post-hoc test analysis was conducted when appropriate. The significance level was set at 5% and SPSS 22 software was used for the analysis.

#### Qualitative analysis

All of the semi-structured interviews were audio-recorded, and then subsequently transcribed. The analysis involved a primarily inductive process of thematic analysis, as described by Braun and Clarke [27]. This was conducted to identify the key themes in the transcriptions and then these themes were considered across each profile. To avoid any potential effects this might have on the inductive process, participants were not aware of which runner category the researchers had classified them into and these categories were not part of the initial stages of analysis. Thematic analysis is a method for identifying, analysing and reporting patterns (or themes) within data. Following the process outlined by Braun & Clarke (2006) [27], six phases of thematic analysis were performed by a trained qualitative researcher (MCDS): 1) familiarising with the data by reading through the entire dataset; 2) generating initial codes including line-by-line coding as needed; 3) searching for themes; 4) reviewing themes; 5) defining and naming themes; 6) producing the finalised analysis and report. During the final stage (stage 6) there was a second layer of analysis included which involved a detailed discussion of how the thematic findings related to each of the running categories defined in the quantitative stage of the project. This involved discussion of the themes produced from the inductive qualitative analysis in terms of the differences and similarities between the different runner categories. As part of quality control, the main two researchers (MB and JL) reviewed the analysis during stage 4 and 5 and confirmed that the results were grounded in the data. Any new findings were included in the analysis.

## Results

### Quantitative results

**Fig 1:**
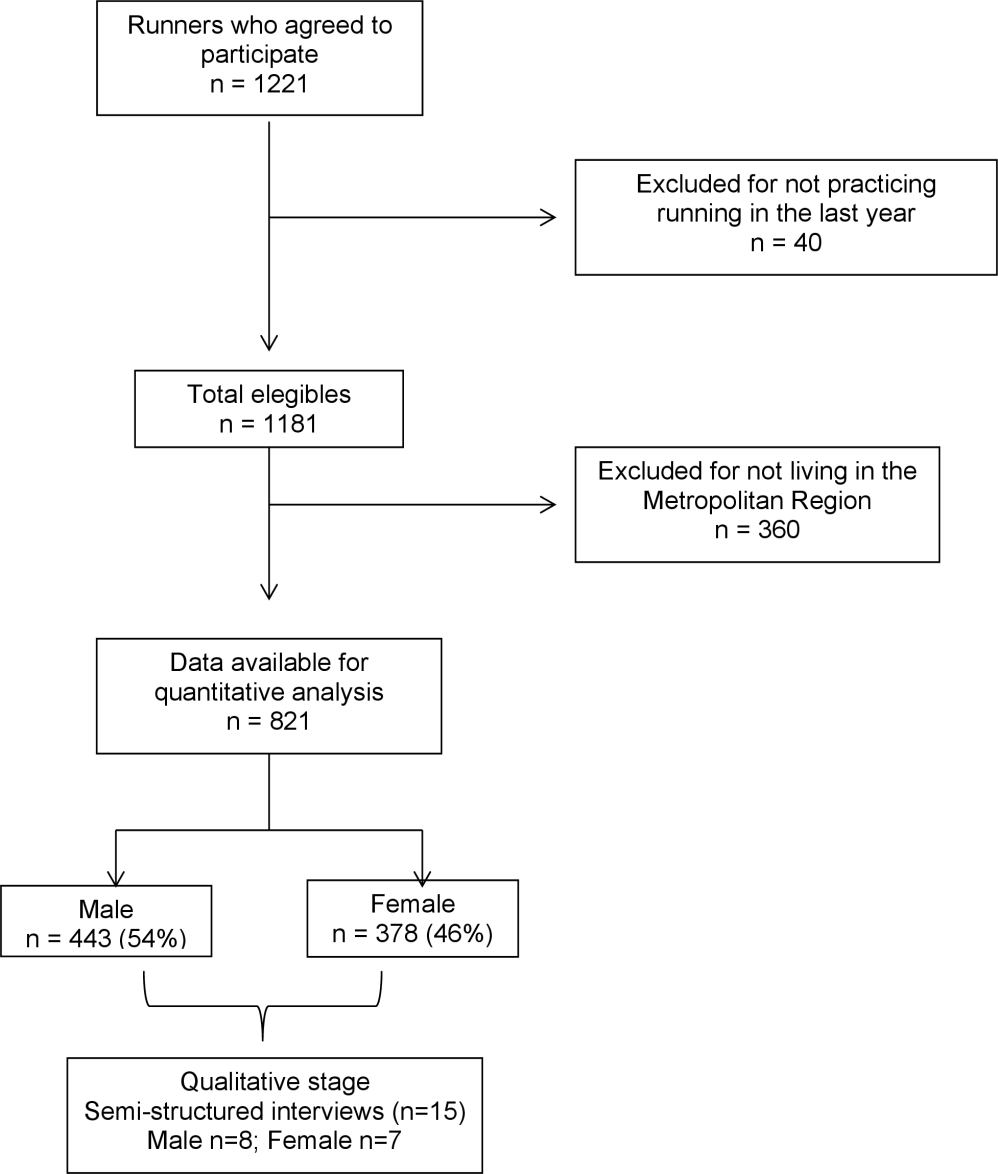
Flowchart of the participant recruitment and selection process.

A total of 1221 participants completed the survey; 821 responses met the eligibility criteria and were analysed. Fig 1 shows a flowchart of the methodological process.

Regarding *socio-demographics and anthropometrics* variables, 443 (54%) were male, with a mean age of 32.6 ± 10 years. On average males and females reported a normal weight by BMI (23.5 *±* 2.8 kg/m^2^). Among all participants, 42% said they had completed university and 64.7% were single. Statistical differences were found for age and BMI between males and females. Table 1 shows socio-demographic and anthropometric characteristics according to sex.

**Table 1:** Socio-demographic and anthropometric characteristics according to sex in SeRUN^®^ study 2015–2016.

		Total	Male	Female
	n	(n = 821)	(n = 443)	(n = 378)
Age (years) ^a^ ^†^	821	32.6 ± 10.0	34.1 ± 10.6	30.7 ± 9.0
BMI (kg/m^2^) ^a^ ^†^	821	23.6 ± 2.8	24.5 ± 2.7	22.6 ± 2.6
Marital status ^b^ ^†^	821			
Single		531 (64.7)	254 (57.3)	277 (73.3)
Married		230 (28.0)	158 (35.7)	72 (19.0)
Divorced or separated		60 (7.3)	31 (7.0)	29 (7.7)
Educational level ^b^	821			
Incomplete university		141 (17.2)	79 (17.8)	62 (16.4)
Complete university		345 (42.0)	173 (39.1)	172 (45.5)
Postgraduate studies		194 (23.6)	114 (25.7)	80 (21.2)
Others (secondary and technical education)		141 (17.2)	77 (17.4)	64 (16.9)

^a^ mean ± standard deviation

^b^ absolute frequency (%). BMI: Body Mass Index.

^†^p-value < 0.05, calculated with t-test and chi-square, as appropriate.

Regarding *training characteristics*, a mean weekly volume of 30.0 *±* 19.7 kilometres was reported, being higher in males (36.1 *±* 21.1 km/week) than females (24.5 *±* 15.5 km/week) (p<0.01). Differences were found (p<0.05) for the average frequency (days/week) between males (3.6 ± 1.4) and females (3.3 ± 1.3), but not for hours of running training (hours/week) reporting 5.4 ± 3.2 hours/week in overall. From all participants, 69.2% reported to use a self-trained plan, 66.7% have complemented their running routine with physical training and 88.2% usually performed stretching exercises.

According to *running experience*, 10.1% said they had less than 6 months practicing the sport, 35.7% between 6 months and 2 years, 20.5% between 3 and 4 years and 33.7% reported 5 years or more. These proportions were different between sex (p<0.05), where males reported the highest proportion for 5 years or more (41.5%) whereas females for less than 1 year (30.7%). Only 9.3% of participants reported not having competitive experience in the last year, while 58.7% of males and 46.1% of females noted participated in five or more previous races, respectively. Most of individuals (78.2%) trained on mixed terrain (hard and soft) and used technological devices such as a mobile application (34.5%) or Global Positioning System (GPS) wristwatch (29.8%) to monitor their training. Besides running, all participants had practiced at least one other sport. Cycling (38.4%), swimming (18.5%), soccer (14.9%) and physical conditioning/fitness exercise (14.6%) were the most common in males. Cycling (37%), physical conditioning/fitness exercise (18%), swimming (13%) and pilates/yoga (9.3%) were most common in females.

Regarding *health-related factors*, most of runners reported consuming between 1 and 2 litres of water per day (65.2%), and a sleeping habit between 6 and 8 hours (70.1%), whereas around 25% of subjects reported to sleep 6 hours or less per night. Of all participants, 43.1% have had a running-related injury in the last 12 months, the most frequent sites were: the ankle and/or foot (35.7%), knee (31.7%) and lower leg (15%). From those with a history of previous injury (n = 354), 36.8% reported suspension of training and rest, 30.4% have had a health professional consultation, and a 26.6% physiotherapy after the injury, from which the majority had finished their treatment (82.6%), with a median of 10 physiotherapy attendances (range 1–40). More than 90% of participants reported to seek a physiotherapist as “useful”.

For the *motivation dimension*, the highest scores—in a 1 to 7 scale—of motivation for running were related to health: “to improve my overall health” (6.0 ± 1.5), to self-esteem: “to feel accomplished and proud of myself; spirit of overcoming” (5.5 ± 1.7), to weight control: “to help controlling my weight (5.4 ± 1.7) and to competitive goals: “to improve my racing time or mark” (5.0 ± 1.8). The lowest score was related to external recognition, reported as “my family and friends are proud of me” (2.7 ± 1.8).

#### Development of running profiles

The cluster analysis allowed the delineation of four profiles or clusters with a total of n = 752 subjects classified (91.6% of the sample). These profiles were formed by the following variables: years of running experience, volume (km/week) and hours (hours/week) of running training. Running volume was adjusted by sex given the significant differences (p<0.05) between males and females. Discriminant analysis showed that 100%, 99.7% and 81.7% of subjects were correctly classified for years of running experience, volume and hours of running training, respectively. Because years of running experience was the main variable that delineated groups, profiles were named according to experience from lowest to highest as: Cluster 1: Beginner (21.7%) with less than 1 year, Cluster 2: Basic (21.8%) between 1–2 years, Cluster 3: Middle (21.3%) between 3–4 years, and Cluster 4: Advanced (35.2%) with 5 or more years.

#### Quantitative comparisons by profile

A comparative analysis of variables between profiles was conducted according to previously reported dimensions. Reported years of running experience was significantly different between profiles (p<0.05). More experienced runners tend to report accumulating more kilometres, running more times and more hours per week than less experienced runners (p <0.05). There were found statistically significant and clinically relevant differences for reported sex, age, years of running experience, volume (km/week), frequency (days/week) and hours (hours/week) of running training, technological devices and previous injury between profiles (Table 2). From those with a history of previous injury, the main taken action after the injury for the Advanced group was to undergo physiotherapy (35.1%), whereas resting was more prevalent for the remaining groups, with 37.5%, 39.4% and 45.3%, for Middle, Basic and Beginner, respectively.

**Table 2:** Profiles characterization according to statistically significant and clinically relevant variables in SeRUN^®^ study 2015–2016.

	Total	*CI (95%)*	Beginner	*CI (95%)*	Basic	*CI (95%)*	Middle	*CI (95%)*	Advanced	*CI (95%)*
	(n = 752)		(n = 163)		(n = 164)		(n = 160)		(n = 265)	
**Sex** ^**a**^ ^**†**^										
Male	417 (55.4)	*51*.*9–59*.*0*	60 (36.8)	*29*.*4–44*.*7*	101 (61.6)	*53*.*7–69*.*1*	83 (51.9)	*43*.*8–59*.*8*	173 (65.3)	*59*.*2–71*.*0*
Female	335 (44.6)	*41*.*0–48*.*1*	103 (63.2)	*55*.*3–70*.*6*	63 (38.4)	*30*.*9–46*.*3*	77 (48.1)	*40*.*2–56*.*2*	92 (34.7)	*29*.*0–40*.*8*
**Age (years)** ^**b**^ ^**†**^	33.0 ± 10.0	*32*.*3–33*.*7*	28.5 ± 8.4	*27*.*2–29*.*8*	31.1 ± 8.6	*29*.*8–32*.*4*	32.4 ± 8.7	*31*.*0–33*.*8*	37.4 *±* 10.9	*36*.*1–38*.*7*
**Running experience** ^**a**^ ^**†**^										
< 6 months	70 (9.3)	*7*.*4–11*.*6*	70 (42.9)	*35*.*2–50*.*9*	-		-		-	
Between 6 months—1 year	93 (12.4)	*10*.*2–14*.*9*	93 (57.1)	*49*.*1–64*.*8*	-		-		-	
Between 1–2 years	164 (21.8)	*19*.*0–24*.*9*	-		164 (100)	*97*.*8–100*	-		-	
Between 3–4 years	160 (21.3)	*18*.*5–24*.*4*	-		-		160 (100)	*97*.*7–100*	-	
Between 5–6 years	97 (12.9)	*10*.*7–15*.*5*	-		-		-		97 (36.6)	*30*.*8–42*.*7*
> = 7 years	168 (22.3)	*19*.*5–25*.*5*	-		-		-		168 (63.4)	*57*.*3–69*.*2*
**Volume (km/week)** ^**b**^ ^**†**^	31.0 ± 19.7	*29*.*6–32*.*4*	18.3 ± 12.7	*16*.*4–20*.*3*	28 ± 17.7	*25*.*3–30*.*7*	35.1 ± 8.6	*32*.*2–38*.*0*	38.2 ± 20.8	*35*.*7–40*.*7*
**Hours (hrs/week)** ^**b**^ ^**†**^	4.4 ± 2.2	*4*.*2–4*.*5*	4.3 ± 2.7	*3*.*9–4*.*7*	4.9 ± 3.2	*4*.*5–5*.*4*	6.0 ± 3.2	*5*.*5–6*.*5*	6.1 ± 3.4	*5*.*7–6*.*5*
**Frequency (day/week)** ^**b**^	3.6 ± 1.3	*3*.*5–3*.*6*	3.1 ± 1.2	*2*.*9–3*.*3*	3.3 ± 1.4	*3*.*1–3*.*5*	3.8 ± 1.3	*3*.*6–4*.*0*	3.9 ± 1.3	*3*.*7–4*.*0*
**Previous injury (12 months)** ^**a**^ ^**†**^	329 (43.8)	*40*.*2–47*.*3*	53 (32.5)	*25*.*4–40*.*3*	83 (55.6)	*42*.*7–58*.*5*	76 (47.5)	*39*.*6–55*.*5*	117 (44.2)	*38*.*1–50*.*4*
**Technological implements** ^**a**^ ^**†**^										
Watch with GPS	318 (42.3)	*38*.*8–45*.*9*	24 (12.7)	*8*.*3–18*.*3*	56 (25.1)	*19*.*6–31*.*3*	87 (37.3)	*31*.*1–43*.*9*	151 (40.8)	*35*.*8–46*.*0*
Watch without GPS	59 (7.9)	*6*.*1–10*.*0*	12 (6.3)	*3*.*3–10*.*8*	22 (9.9)	*6*.*3–14*.*6*	13 (5.6)	*3*.*0–9*.*4*	17 (4.6)	*2*.*7–7*.*3*
Heart rate monitor	27 (3.6)	*2*.*5–5*.*2*	19 (10.1)	*6*.*2–15*.*3*	26 (11.7)	*7*.*8–16*.*6*	39 (16.7)	*12*.*2–22*.*2*	77 (20.8)	*16*.*8–25*.*3*
Mobile application	229 (30.4)	*27*.*3–33*.*8*	86 (45.5)	*38*.*3–52*.*9*	95 (42.6)	*36*.*0–49*.*4*	85 (36.5)	*30*.*3–43*.*0*	87 (23.5)	*19*.*3–28*.*1*
None	119 (15.8)	*13*.*4–18*.*6*	48 (25.4)	*19*.*4–32*.*2*	24 (10.8)	*7*.*0–15*.*6*	9 (3.9)	*1*.*8–7*.*2*	38 (10.3)	*7*.*4–3*.*8*

^a^ Absolute frequency (%)

^b^ mean ± standard deviation

BMI: Body Mass Index. CI: Confident Interval (95%); km/week: kilometres per week; day/week: days per week; hrs/week: hours per week; GPS: Global Positioning System.

^†^p-value < 0.05 (chi-square^a^ or ANOVA^b^, as appropriate).

Motivations for running were similar to those found in the general population analysis. However, there were differences identified (p<0.05) for the mean score of competitive goals dimension between Advanced (5.3 ± 1.6), Middle (5.2 ± 1.8) and Basic (5.2 ± 1.7) groups with Beginner (4.6 ± 2.1); and for affiliation/social dimension between Advanced (4.2 ± 1.9) and Beginner groups (3.6 ± 2.0).

**Fig 2:**
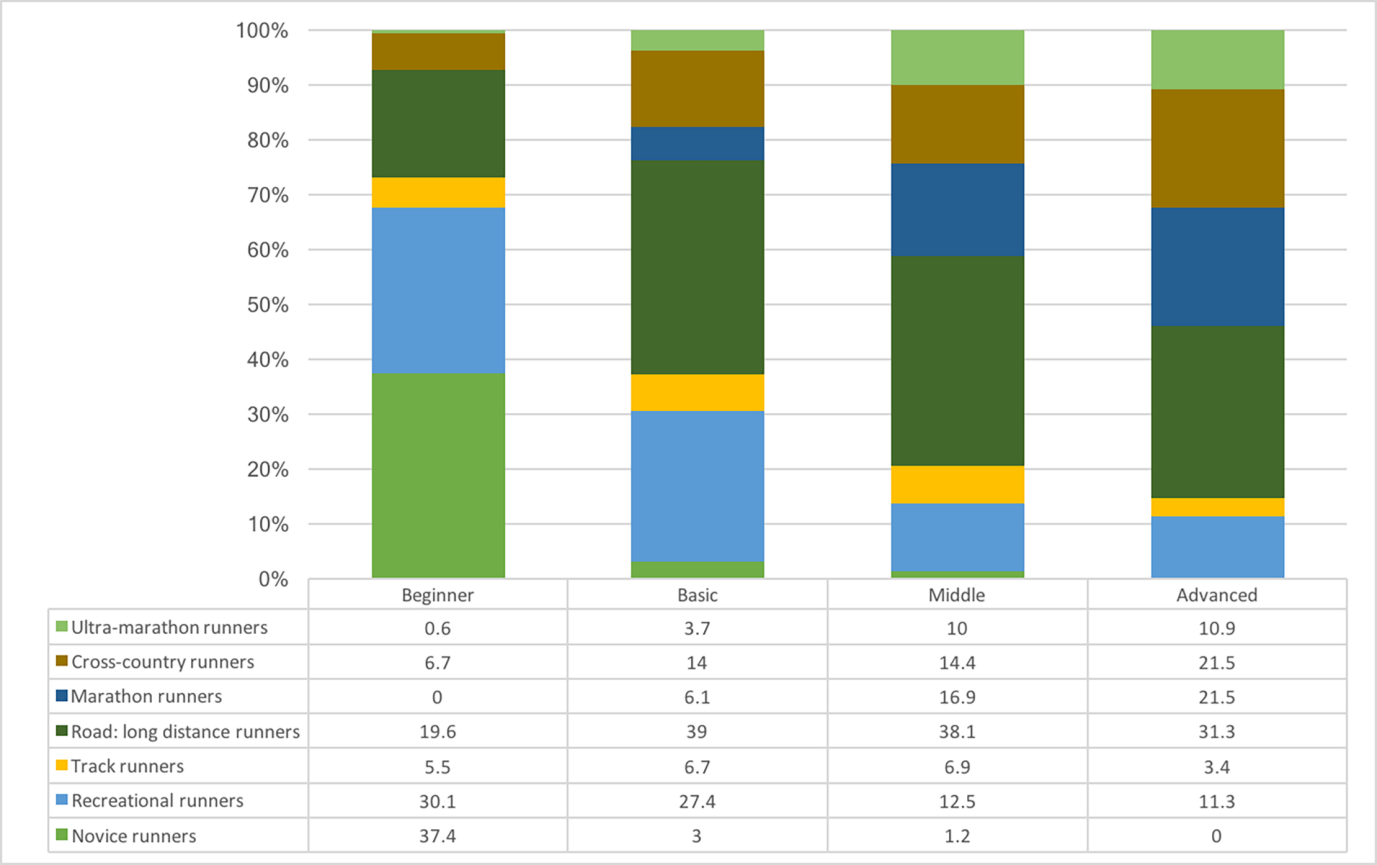
Graphical distribution of Kluitenberg’s classification by profile.

According to the classification described by Kluitenberg et al. [7], runners classified themselves in one of the seven categories presented, where the largest proportions were found for long distance (30.4%) and recreational runners (21%). The distribution of running profiles across the seven categories is included as supplementary material (S1 Fig). When analysed by profiles, Beginner runners classify themselves mainly as novice (37.4%) and recreational runners (30.1%); Basic as road long distance (39%) and recreational runners (27.4%); Middle as road long distance (38.1%), marathon (16.9%) and cross-country runners (14.4%); and Advanced as road long distance (31.3%), marathon (21.5%) and cross-country runners (21.5%). The graphical distribution of Kluitenberg’s classification by profile is shown in Fig 2.

### Qualitative results

Qualitative data analyses allowed insight into the reasons behind some of the quantitative results regarding the following themes: 1) different motivations to start and keep running; 2) beliefs about injury risk factors and injury prevention; and 3) the role of the physiotherapist in the rehabilitation process for running-related injuries. We discuss each of these themes in relation to the runner profiles *Beginner*, *Basic*, *Middle* and *Advanced*. See Table 3 for a summary of both qualitative and quantitative results according to runner profile.

**Table 3:** Summary of quantitative and qualitative results according to SeRUN^®^ profiles 2015–2016.

DIMENSIONS	Beginner	Basic	Middle	Advanced
	(n = 163)	(n = 164)	(n = 160)	(n = 250)
**1) Socio-demographic**	60 (36.8)	101 (61.6)	83 (51.9)	173 (65.3)
• Gender (male) ^a^	28.5 ± 8.4	31.1 ± 8.6	32.4 ± 8.7	37.4 *±* 10.9
• Age (years) ^b^				
**2) Health**				
• Previous RRI ^a^	53 (32.5)	83 (55.6)	76 (47.5)	117 (44.2)
• Physical Therapy after RRI ^a^	14 (15.9)	38 (26.2)	31 (29)	80 (34.6)
**3) Motivation** ^**c**^	Accessibility and low cost; Health and lifestyle/weight; Psychological features	Accessibility and low cost; Health and lifestyle/weight; Psychological features	Accessibility and low cost; Health and lifestyle/weight; Affiliation/team; Competition; Psychological features	Accessibility and low cost; Health and lifestyle; Affiliation/team; Competition; Psychological features
**4) Behaviour associated with running**				
• Years of running experience	< 1	1–2	3–4	> 4
• > 4 running races ^a^	46 (28.4)	100 (61.7)	110 (68.8)	160 (60.6)
**5) Training**				
• Volume (km/week) ^b^	18.3 ± 12.7	28 ± 17.7	35.1 ± 18.6	38.2 ± 20.8
• Frequency (days/week) ^b^	3.1 ± 1.2	3.3 ± 1.4	3.8 ± 1.3	3.9 ± 1.3
**6) Beliefs/perceptions about health**				
• Causes of RRI ^c^	Overload/over-exertion, footwear	Overload/over-exertion, footwear, training surface	Overload/over-exertion, running technique, footwear, physical preparation	Overload/over-exertion, running technique, footwear, physical preparation
• Physiotherapist role ^c^	Specialist, involved in process, empathic	Specialist, involved in process, empathic	Specialist, involved in process, empathic	Specialist, involved in process, empathic

^a^ Absolute frequency (%)

^b^ mean ± standard deviation

^c^ thematic analysis.

RRI = Running-Related Injury; km = kilometres

#### Motivation to start running and reasons for permanence

Similar reasons were reported as driving motivation to start running *across the four profiles*. Ease of practice, saving time and low cost were the main reasons: “It’s easier to start compared to other physical activities, it’s accessible” (Case 1, Advanced profile); “It is an easy sport, compared with any other sport running it is very cheap” (Case 7, Middle profile); and “You can just have simple shoes and go out and run on the street, in a park or anywhere” (Case 13, Beginner profile).

Participants *across all profiles* gave health-related reasons for continuing to run (permanence). They considered running to be part of a lifestyle that brings health benefits, as well as a method to manage and reduce daily stress. Participants reflected this as “Having a healthy lifestyle, moving around” (Case 3, Advanced profile); “Get out of society’s routine a little bit” (Case 5, Middle profile); and “This is like my best motivation, the quality of life” (Case 11, Basic profile). However, other reasons for continuing to run were different across the categories:

1. *Beginner* and *Basic*: Driving motivations of improving personal appearance and aesthetics were mainly addressed by participants within these profiles: “There is an interest in improving health, lose weight, and feeling healthy and active” (Case 8, Beginner profile); and “…you can see results on your body” (Case 11, Basic Profile).
2. *Middle* and *Advanced*: Achieving competitive goals and connection with nature were the main motivations for participating runners within these profiles: “Personally, the motivation is clearly the connection with nature” (Case 5, Middle profile); and “It is like when you reach certain distances, OK, you start asking yourself ‘if I’ve reached this point, am I able to do more?” (Case 6, Advanced profile).

Participants *across all profiles* also discussed that addiction can be part of the drive to run, which is less present when beginning, but later becomes a large aspect over time: “Little by little it started to grab me, which tends to happens … but it’s one of those things that can suddenly hook you, doesn’t let you go” (Case 1, Advanced profile); "When I discovered it I absolutely loved it, I was fascinated and I would say that it has been one of the few things in life that I have been so passionate about" (Case 7, Middle profile).

Psychological and social features were reported by *all profiles*. Psychological aspects included self-connection, meditation, and happiness: "Running makes you have a connection with yourself" (Case 12, Beginner profile). More experienced runners (*Middle* and *Advanced*) additionally reported being involved in teams or groups as a positive social experience which promoted training and participation in competition: “So it is not only training with another person, it is training with a friend, with a teammate until the end. Even planning races together” (Case 5, Middle profile). Benefits were reported regarding support and orientation towards practicing the sport appropriately: “In some moment of your running life, you need the feedback of a male or female teammate and it is nice to share tips and info, you go clearing up lots of doubts, a lot of benefits, you get rid of certain anxieties regarding others’ experiences” (Case 2, Advanced profile). The opposite was found in less experienced runners (*Basic and Beginner*): “It is cool to have a running group and everything, but sometimes they tell you that ‘these days a week’ you have to go here or there. I prefer to go out when I want and without anyone telling me a certain hour” (Case 11, Basic profile). Indeed, one female runner preferred aloneness to group practice: “No, to me it is too much; when I run with others I can’t stay in my pace, and if I want to just stop and do some push-ups I don’t have to wait around for anyone” (Case 14, Basic profile).

#### Beliefs about risk factors for running-related injuries

Interviewees *across all profiles* identified the concept of running-related injury as pain induced by running: “They are the ones that start after running; you notice that after you run, different aches start, and they are typical aches and pains, like the sole of the foot, the knee and ankle” (Case 4, Advanced profile); “It would be like an injury provoked from running” (Case 7, Middle profile).

Seeking health professionals too late or running in spite of feeling pain, were general behaviours reported by *all runners*: “When I feel like it really, really hurts, I mean really hurts, I’m kind of stubborn to go to the doctor” (Case 4, Advanced profile). Another expressed: “For example, I had that pain in my ankle, I mean more than a little pain it was a huge suffering and a giant pain, I didn’t go in to have it checked at the beginning, I just kept running” (Case 11, Basic profile).

*Across all profiles*, runners reported overload or fatigue as a risk factor: “Some (injuries) are the product of fatigue, I mean, when one runs a lot, of course you’re tired and probably sore, I don’t know, but that’s just small pain, not an injury at the end. I mean it’s something that goes away with running over time and training” (Case 1, Advanced profile). “Also I over-trained sometimes, I just ran ran ran and felt a little pain, and go go go, and I kept on week after week after week until it finally caught up to me” (Case 8, Basic profile).

Additional risk factors were by *all runners regardless of profile*, but the more experienced runners identified more factors. Beginners identified footwear, whereas Basic runners identified both footwear and training surface, and more experienced runners discussed both footwear and surface but also added lack of physical preparation:

1. *Beginner*: **a)** inappropriate footwear: “With a bad shoe—as I imagine—the menisci can have a strong impact when you are stepping or running, as the vibrations are a bit strong” (Case 14).
2. *Basic*: **a)** inappropriate footwear: “…and then I learned that there were special running shoes that had better cushioning" (Case 9); **b)** training surface: “I got shin splints in the shins, that’s what they call them, shins. Because of the type of asphalt, I mean the asphalt is really hard” (Case 8).
3. *Middle and Advanced*: ***a)*** inappropriate footwear: “If, for example, the biomechanics of your foot, OK, implies that you should use some type of shoe in particular and if you do not use it, you will get injured” (Case 6); **b)** running technique: "I have also noticed things that I did not realize before; I have tried to improve postures, as I said before, that not many know about the running technique or the correct postures” (Case 5); **c)** lack of preparation: “The irresponsibility of getting me into a longer career without being prepared” (Case 1).

Recommendations for injury management and prevention varied between less and more experienced runners:

1. *Beginner* and *Basic*: **a)** rest, “More than anything it was a lot of resting and stopping running” (Case 8, Basic profile); **b)** use of appropriate footwear, “After the injury I changed shoes because the physical therapist told me that the shoes have to be with better cushioning, or they had to be a little higher and those shoes were better” (Case 9, Basic profile); **c)** stretching and other habits: “Stretch well, eat well, hydrate well, not over exert yourself, and that, have fun, listen to music, that’s it.” (Case 11, Basic profile).
2. *Middle* and *Advanced*: **a)** running technique, “If you do not have a running technique, you are going to get hurt” (Case 6, Middle profile); **b)** physical preparation: “You have to prepare the body to avoid having injuries” (Case 6, Middle profile); **c)** releasing techniques: “There are things that I know now, that I detect, so I know I have to stretch more, I know for example that I have to freeze bottles of water, and roll my feet over them (Case 3, Advanced profile); **d)** incorporate trail running into the routine: “Trail running helps you in the prevention of injuries. The asphalt is so hard for the joints, all the time hitting the same side” (Case 7, Middle profile).

#### Physical therapist role

No differences were found among profiles related to the role of the physiotherapist. The physiotherapist was considered a useful professional in the injury rehabilitation process *across all profiles*, identifying common treatments as electrotherapy, cold/heat therapy, massage and exercise. “It is a necessary evil, if you’re complaining of an injury there is not even any other option except for a good physical therapy session, and that means that usually it’s not less than 10 sessions, where you have to concentrate your strength on recovering … in physical therapy terms with ultrasounds, massages and that stops you from running, because if you run you’re going to re-injured yourself, and after all it’s worse, you have to respect the physical therapy time, no matter what” (Case 2, Advanced profile).

Participants in *Advanced*, *Middle and Basic profiles* agreed that it is recommended to seek professional guidance when facing a prolonged injury, especially a specialist in sports injuries. The importance of acting responsibly when instructed by such specialist was also highlighted by participants: “You have to go to a physical therapist, expert in sports, and hopefully in the injured area, which is the same as traumatology, they are divided by different problems, you have to look for the physical therapist that is precisely dedicated to sports and the type of injury that you have, make sure he knows that part of the body” (Case 4, Advanced profile). Another participant expressed, “From the beginning it has to be someone specialized in sports, who knows what we’re talking about, the thing about stride, about the person … if they are running ten kilometres, twenty-kilometres or if the person runs hills or run on concrete all the time, it’s different” (Case 9, Basic profile).

Participants across *all profiles* highlighted that a psychological approach was an important role for physical therapists to adopt in the rehabilitation process, taking a paternalistic role and being involved in the whole process. For example, one participant said:

The physical therapist should be a person that is primarily a psychologist, because you are there with an injury and you’re affected, afraid, anxious, and physical therapist has to calm you down, he has to hear you and be with you in the whole process of rehabilitation. There are some physical therapists where you go, they put you in the box with the machine, and that’s it. A good physical therapist, different from a mediocre one, is one who is involved in the whole process of recuperation along with you (Case 2, Advanced profile).

The physiotherapist has to have the capacity to listen and empathize with the patient, “That they listen to what the person needs specifically, sometimes they just have their own ideas about what the person needs, and it’s not what the person wants” (Case 3, Advanced profile).

## Discussion

The most important finding of this study was the identification of four running profiles, defined by years of experience, accumulated weekly volume (km/week) and hours of training (hours/week) based on a statistic model through a cluster analysis. Qualitative information further explored the characterization of runners, particularly pertaining to the dimensions of motivations and beliefs about health, paying special attention to injuries, risk factors and how to prevent them.

Currently, the study of aetiology and treatment of RRIs is the major focus on running research [28, 29]. SeRUN^®^ profiles allow an appropriate classification for the recruitment process and therefore, a potential control of confounding factors [30, 31], such as previous injury, running volume and behaviours associated with its practice [13] (Table 3). The comparison of confidence intervals among profiles (Table 2) indicates that years of running experience and training volume (km/week) are clearly delineated within each of the running profiles, except for training volume between Middle and Advanced profiles. These findings suggest that training volume is likely to be accurate to categorise runners and help to explain the classification based on running experience. Indeed, a design protocol study was currently published (ProjectRun21) [32] with the purpose of examine if particular subgroups of half-marathon runners are more likely to sustain a RRI based training factors and running experience.

These profiles are not exempt from the self-classification of the type of runner (e.g. recreational, long distance runner, cross-country) [7], evidenced in the wide variability found (Fig 2 and S1 Fig). Most running research includes recreational and novice runners as the study population [2, 7, 33], without a specific definition and based on the ‘regular’ participation in running. According to this study, a recreational runner may or may not have competitive motivations, with a high or very low running experience, be female or male. These factors highlight the need for a clear definition of what a recreational runner is.

The anthropometric, health and training characteristics of this sample were similar to those reported from other Latin American populations such as Brazil [34]. Regarding behaviours associated with running practice, results showed higher level of running experience compared to the general population [34–36], with more than half reporting at least three years of running experience and five previous participations in competition. Even though the main proportion of novice runners were classified as Beginners (having less than 1 year of running experience), it is possible that a proper group of ‘novices’ was not recruited, due to their irregular experience or how they classify themselves as runners.

Previous literature has shown that previous injury, training volume and running experience are characteristics associated with RRIs [9, 12, 13] in general running populations. Considering risk factors, the U–inverted pattern of previous injury reporting might reflect that those factors are differently distributed between profiles. Future studies should analyse these factors, incorporating anatomical, biomechanics, motor control, social and psychological measurements that may be related to injury development. The lowest proportion of previous injury in Beginners (32.5%) may indicate a window of opportunity for intervention, assuming a linear growth of a runner’s experience. Other studies are required, preferably longitudinal, that identify the evolution of a runner with respect to their musculoskeletal health and injury development.

The main reasons reported as being most relevant for developing a RRI (Table 3) were similar to those found in the literature for more experienced groups [37], identifying factors such as training and physical preparation, which might be beliefs created by the experience. Although care should be taken when interpreting these results because only recreational runners were recruited in Saragiotto’s study. On the other hand, less experienced groups (Beginner and Basic) had higher responses for stretching, footwear and training surface which are secondary factors to what is generally reported [13, 38–40] and might be beliefs created by the environment and marketing. Educational strategies around these findings could be developed, and modified accordingly in order to more effectively target specific populations of runners and their beliefs. The habit of prolonging training while suffering from an injury should be a general focus point for education across all profiles of runners.

Runners who suffer a RRI do not tend to seek a health professional right away. Later a learning phenomenon appears to develop as a product of the rehabilitation process, as runners learn different care strategies. Another important finding is the belief about the role of the physical therapist in the rehabilitation process. Runners identified that they believe the physical therapist should be a specialist, involved in the process and showing empathy towards the patient, showing a paternalistic role. In contrast to current recommendations [41], the role of the physical therapist was not seen as preventative, nor was it associated with a multidisciplinary team of professionals, such as coaches, physical trainers, or nutritionists.

The primary reported motivation for running was related to health, which is different from what is reported in other literature. Motivation has previously been shown to shift in the life of a runner, from goal achievement to psychological wellbeing [42], which were found as main motivations for more experienced runners. The motivations reported in the current study might have been affected by the study design, with a two-stage explanatory approach. Similar to the results from this study, Ogles et al. [43] concluded that women’s motivation to run were more related to their weight, affiliation, psychological goals and a meaningful purpose in life; whereas males stated that their main motivations to run were based on achievements such as competitions and personal goals. Future research should incorporate assessment of psychological outcomes that may be associated with health-related behaviours of runners [14].

The incorporation of new paradigms for approaching health problems, such as using mixed methods (quantitative and qualitative), allows a more global understanding of the problem [17, 18], involving participant perspectives into the study process. Knowing the necessities of runners and considering their health constructs, could help to generate educational programs specifically targeted to their beliefs [30].

### Study limitations

Principal limitations of this study are related to the measurement instrument employed–data was all self-reported therefore while it is likely to bear a relationship to what happens in reality, certain elements may be over- or under-reported. It is recommended for future research to incorporate objective measurements especially for training variables, such as weekly running volume, intensity and type of training [44]. In addition, the convenience sample used for both stages might have introduced selection bias. Even though relative saturation was reached in the qualitative stage, as themes had sufficient data for analysis, the analysis by profile might be improved with a larger sample size.

## Conclusions

Running profiles were delineated according to years of running experience, accumulated weekly volume and hours of training, where the primary variable that allowed the classification of subjects was years of experience. Qualitative results added further richness, depth of understanding, and possible new parameters for consideration. Subjects with more experience tended to accumulate a higher weekly training load (kilometres and hours), being older, have a higher proportion of previous injury, use more specialized equipment to monitor their training and have experience-based beliefs about self-care in health, comparing to less experienced runners.

### Implications for clinical practice

Incorporation of adequate evaluation systems that identify susceptible subjects and in risk of injury is necessary for managing RRIs. Training load is a factor that should be incorporated by health professionals and trainers when evaluating, treating and training runners, as well as their knowledge about musculoskeletal health. It is recommended that the experience level of runners and their health-related beliefs are considered to properly develop educational strategies, understanding that running injuries follow a multi-causal pattern.

### Implications for research

The present study is the (known) first investigation within the health field that classifies and identifies profiles or types of runners using a mixed model and a common classification criterion (SeRUN^®^ profile). The identification of these profiles might facilitate an appropriate and accurate recruitment process for future prospective studies and clinical trials that evaluate risk and prognostic factors for each group, as well as treatment and injury prevention programs.

## Supporting information

S1 Fig Graphical distribution of running profiles, according to Kluitenberg's classification in SeRUN^®^ study 2015–2016. This graph contains the percentage distribution of running profiles for each category described in Kluitenberg et al [7]: novice runners, recreational runners, track runners, road: long distance runners, marathon runners, cross-country runners and ultra-marathon runners.
(TIFF)

S1 File Outline form for semi-structured interviews. This file contains the 14 questions used to guide the semi-structured interview. It was grouped into four sections: 1) introduction: 2) running practice; 3) motivations for running; and 4) health/lifestyle and physiotherapist role.
(PDF)

S2 File Consolidated criteria for reporting qualitative studies (COREQ): 32-item checklist. This file contains the checklist for reporting qualitative research. It has the specific pages were information can be found. Adapted from: Tong A, Sainsbury P, Craig J. Consolidated criteria for reporting qualitative research (COREQ): a 32-item checklist for interviews and focus groups. *International Journal for Quality in Health Care*. 2007. Volume 19, Number 6: pp. 349–357.
(PDF)
