# Supplementary figures and images for: SeRUN® study: Development of running profiles using a mixed methods analysis
Source: PLoS One. 2018 Jul 10;13(7):e0200389. doi: 10.1371/journal.pone.0200389 (PMC6039021; doi:10.1371/journal.pone.0200389)

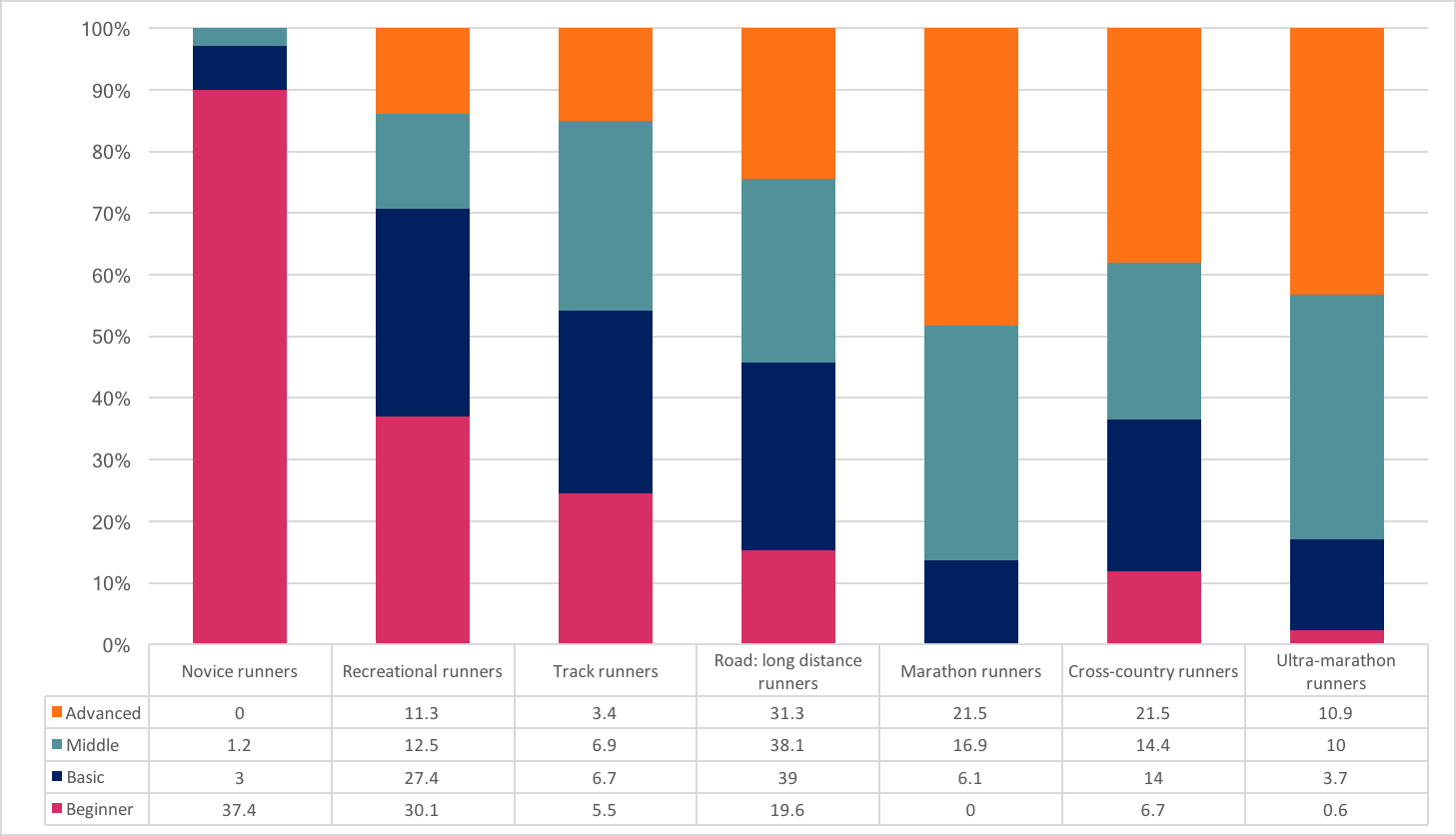

Supplement: S1 Fig — This graph contains the percentage distribution of running profiles for each category described in Kluitenberg et al [7]: novice runners, recreational runners, track runners, road: long distance runners, marathon runners, cross-country runners and ultra-marathon runners. (TIFF) [file pone.0200389.s001.tiff]
