# Supplementary material for: SeRUN® study: Development of running profiles using a mixed methods analysis
Source: PLoS One. 2018 Jul 10;13(7):e0200389. doi: 10.1371/journal.pone.0200389 (PMC6039021; doi:10.1371/journal.pone.0200389)
Supplement: S1 File — This file contains the 14 questions used to guide the semi-structured interview. It was grouped into four sections: 1) introduction: 2) running practice; 3) motivations for running; and 4) health/lifestyle and physiotherapist role. (PDF) [file pone.0200389.s002.pdf]

I. INTRODUCTION. GENERAL SCENERY

1. According to recent data, there has been a boom in running practice in Santiago. What do you think is due?
2. How would you characterize a runner from Santiago? What would be their qualities and / or attributes?

II. RUNNING PRACTICE

3. Tell me about your experience in running. How many years have you been training?
4. ¿How would you describe your training routine: you usually go out running at specific times or days, do you have some kind of special preparation? (Inquire about training plan, where to run, how much, nutritional plan, use of special footwear, etc.)
5. Do you run with a group or are you associated with a team of runners? (Investigate in sociability associated with practice, networks)
6. Did you run in the Santiago 2015 Marathon? How did you prepare? Will you participate in the next Marathon? How are you preparing for?

III. MOTIVATION FOR RUNNING

7. Why did you start running at the beginning? What are the reasons why you run daily? (Investigate different types of motivation)

8. What do you think are the main limitations or barriers and facilities associated with the practice of running?

9. In general, what are your goals or expectations as a future runner?

IV. HEALTH / LIFESTYLE AND PHYSIOTHERAPIST ROLE

10. What do you usually do when you have a discomfort during or after your workouts?

11. What do you understand by running-related injury? Have you had an injury of this type? (Inquire into causes of injury)

12. When and how do you make the decision to visit a health professional? (If so) Whom do you consult in the first instance?

13. Have you attended physiotherapy sessions for any injury? How useful do you think it is to seek a physiotherapist? (It is sought to investigate around the perceptions associated with physiotherapy attention, valuations, appraisals about the utility, etc.)

14. Specifically referring to the work of the physiotherapist. How do you think he/she should focus or guide the work to have more impact?
