# Supplementary material for: SeRUN® study: Development of running profiles using a mixed methods analysis
Source: PLoS One. 2018 Jul 10;13(7):e0200389. doi: 10.1371/journal.pone.0200389 (PMC6039021; doi:10.1371/journal.pone.0200389)
Supplement: S2 File — This file contains the checklist for reporting qualitative research. It has the specific pages were information can be found. Adapted from: Tong A, Sainsbury P, Craig J. Consolidated criteria for reporting qualitative research (COREQ): a 32-item checklist for interviews and focus groups. International Journal for Quality in Health Care. 2007. Volume 19, Number 6: pp. 349–357. (PDF) [file pone.0200389.s003.pdf]

Consolidated criteria for reporting qualitative studies (COREQ): 32-item checklist

Adapted from:
Tong A, Sainsbury P, Craig J. Consolidated criteria for reporting qualitative research (COREQ): a 32-item checklist for interviews and focus groups. *International Journal for Quality in Health Care*. 2007. Volume 19, Number 6: pp. 349 – 357

No. Item	Guide questions/description	Comment
**Domain 1: Research team and reflexivity**		
Personal Characteristics		
1. Interviewer/facilitator	Which author/s conducted the interview or focus group?	Page 7 and 8
2. Credentials	What were the researcher's credentials? E.g. PhD, MD	Manuela Besomi (MD), Jaime Leppe (MD), Cristina Di Silvestre (MD), Jenny Setchell (PhD).
3. Occupation	What was their occupation at the time of the study?	Page 8
4. Gender	Was the researcher male or female?	Page 8
5. Experience and training	What experience or training did the researcher have?	Sociologists working on an agency of strategic communication, studies and social research (named Ekhos).
Relationship with participants		
6. Relationship established	Was a relationship established prior to study commencement?	Page 7 and 8
7. Participant knowledge of the interviewer	What did the participants know about the researcher? e.g. personal goals, reasons for doing the research	Participants knew that interviewer was undertaking the interviews for the study and who the lead research was.
8. Interviewer characteristics	What characteristics were reported about the interviewer/facilitator? e.g. Bias, assumptions, reasons and interests in the research topic	Page 8

**Domain 2: study design**		
Theoretical framework		
9. Methodological orientation and Theory	What methodological orientation was stated to underpin the study? e.g. grounded theory, discourse analysis, ethnography, phenomenology, content analysis	Page 9 and 10
Participant selection		
10. Sampling	How were participants selected? e.g. purposive, convenience, consecutive, snowball	Page 6 and 7
11. Method of approach	How were participants approached? e.g. face-to-face, telephone, mail, email	Page 6 and 7
12. Sample size	How many participants were in the study?	Page 6 and 7
13. Non-participation	How many people refused to participate or dropped out? Reasons?	All contacted participants (n=15) agreed to participate
Setting		
14. Setting of data collection	Where was the data collected? e.g. home, clinic, workplace	Page 8
15. Presence of non-participants	Was anyone else present besides the participants and researchers?	No one else was present.
16. Description of sample	What are the important characteristics of the sample? e.g. demographic data, date	From page 10-13
Data collection		
17. Interview guide	Were questions, prompts, guides provided by the authors? Was it pilot tested?	Page 7 (S1 File)
18. Repeat interviews	Were repeat inter views carried out? If yes, how many?	No repeat interviews
19. Audio/visual recording	Did the research use audio or visual recording to collect the data?	Page 9

20. Field notes	Were field notes made during and/or after the interview or focus group?	No field notes
21. Duration	What was the duration of the interviews or focus group?	Page 8
22. Data saturation	Was data saturation discussed?	Not discussed
23. Transcripts returned	Were transcripts returned to participants for comment and/or correction?	No return of transcripts
**Domain 3: analysis and findings**		
Data analysis		
24. Number of data coders	How many data coders coded the data?	N/A
25. Description of the coding tree	Did authors provide a description of the coding tree?	N/A
26. Derivation of themes	Were themes identified in advance or derived from the data?	Page 9 and 10
27. Software	What software, if applicable, was used to manage the data?	N/A
28. Participant checking	Did participants provide feedback on the findings?	No checking of findings by any participants
Reporting		
29. Quotations presented	Were participant quotations presented to illustrate the themes/findings? Was each quotation identified? e.g. participant number	From page 17-23
30. Data and findings consistent	Was there consistency between the data presented and the findings?	From page 17-23
31. Clarity of major themes	Were major themes clearly presented in the findings?	Page 24
32. Clarity of minor themes	Is there a description of diverse cases or discussion of minor themes?	N/A because of the mixed method approach
